# Genetic analysis of phytoene synthase 1 (*Psy1*) gene function and regulation in common wheat

**DOI:** 10.1186/s12870-016-0916-z

**Published:** 2016-10-21

**Authors:** Shengnan Zhai, Genying Li, Youwei Sun, Jianmin Song, Jihu Li, Guoqi Song, Yulian Li, Hongqing Ling, Zhonghu He, Xianchun Xia

**Affiliations:** 1 Institute of Crop Science, National Wheat Improvement Center, Chinese Academy of Agricultural Sciences (CAAS), 12 Zhongguancun South Street, Beijing, 100081 China; 2 Crop Research Institute, Shandong Academy of Agricultural Sciences, 202 Gongye Bei Road, Jinan, Shandong 250100 China; 3 State Key Laboratory of Plant Cell and Chromosome Engineering, Institute of Genetics and Developmental Biology, Chinese Academy of Sciences, Beijing, 100101 China; 4 International Maize and Wheat Improvement Center (CIMMYT) China Office, c/o CAAS, 12 Zhongguancun South Street, Beijing, 100081 China

**Keywords:** Carotenoid biosynthesis, RNAi, RNA-Seq, TILLING, *Triticum aestivum*

## Abstract

**Background:**

Phytoene synthase 1 (PSY1) is the most important regulatory enzyme in carotenoid biosynthesis, whereas its function is hardly known in common wheat. The aims of the present study were to investigate *Psy1* function and genetic regulation using reverse genetics approaches.

**Results:**

Transcript levels of *Psy1* in RNAi transgenic lines were decreased by 54–76 % and yellow pigment content (YPC) was reduced by 26–35 % compared with controls, confirming the impact of *Psy1* on carotenoid accumulation. A series of candidate genes involved in secondary metabolic pathways and core metabolic processes responded to *Psy1* down-regulation. The aspartate rich domain (DXXXD) was important for PSY1 function, and conserved nucleotides adjacent to the domain influenced YPC by regulating gene expression, enzyme activity or alternative splicing. Compensatory responses analysis indicated that three *Psy1* homoeologs may be coordinately regulated under normal conditions, but separately regulated under stress. The period 14 days post anthesis (DPA) was found to be a key regulation node during grain development.

**Conclusion:**

The findings define key aspects of flour color regulation in wheat and facilitate the genetic improvement of wheat quality targeting color/nutritional specifications required for specific end products.

**Electronic supplementary material:**

The online version of this article (doi:10.1186/s12870-016-0916-z) contains supplementary material, which is available to authorized users.

## Background

Carotenoids, a complex class of C40 isoprenoid pigments synthesized by photosynthetic organisms, bacteria and fungi [1], are essential components of the human diet. The most important function is as a dietary source of provitamin A [2]. Vitamin A deficiency can result in xerophthalmia, increased infant morbidity and mortality, and depressed immunological responses [3]. Additionally, carotenoids as antioxidants can reduce the risk of age-related macular degeneration, cancer, cardiovascular diseases and other chronic diseases [4]. Common wheat (*Triticum aestivum* L.) is a major cereal crop, supplying significant amounts of dietary carbohydrate and protein for over 60 % of the world population. It is also an important source of carotenoids in human diets [5]. Moreover, carotenoids in wheat grains determine flour color, an important quality trait for major wheat products such as noodles.

Phytoene synthase (PSY) catalyzes a vital step in carotenoid biosynthesis, generally recognized as the most important regulatory enzyme in the pathway [1, 6]. Although there are up to three PSY isozymes in grasses, only *Psy1* expression is associated with carotenoid accumulation in grains [7, 8]. The wheat *Psy1* gene was cloned based on the sequence homology, and QTL analysis showed that *Psy1* co-segregated with yellow pigment content (YPC), which is significantly related to carotenoids (*r =* 0.8) [6, 9]. To date, several studies have focused on homology-based cloning of *Psy1* and QTL analysis, whereas gene function and regulation remain to be determined.

Common wheat has a large genome that consists of three closely related (homoeologous) genomes with 93–96 % sequence identity and a high proportion of repetitive sequences (>80 %) [10]. Homoeologous gene duplication limits the use of forward genetics due to compensatory processes that mask the effects of single-gene knockout mutations [11]. Therefore, the ability to investigate gene function and regulation in wheat ultimately depends on robust, flexible, high-throughput reverse genetics tools.

RNA interference (RNAi) is a sequence-specific gene suppression system that has been used in a variety of plant species as an efficient tool to decrease or knock-out gene expression. RNAi has an enormous potential in functional genomics of common wheat, because all homoeologs (from the A, B and D subgenomes) can be simultaneously silenced by a single RNAi construct [12]. To date, RNAi has been used to target a wide range of genes in wheat, including those encoding lipoxygenase, starch biosynthetic enzymes, and proteins involved in storage [13–15].

With next-generation high-throughput sequencing technologies, RNA-sequencing (RNA-Seq) has emerged as a useful tool to profile genome-wide transcriptional patterns in different tissues and developmental stages, and can lead to the discovery novel genes in specific biological processes [16]. In this context, comparative analysis of transcriptome data between transgenic lines and wild type can reveal the transcriptional regulation network associated with genetic change.

Targeting induced local lesions in genomes (TILLING) is a powerful reverse genetics approach combining chemical mutagenesis with a high-throughput screen for mutations, and has been widely used in functional genomics [17]. Compared to typical reverse genetics techniques such as RNAi and insertional mutagenesis, the main advantage of TILLING is the ability to accumulate a series of mutated alleles, including silent, missense, truncation or splice site changes, with a range of modified functions, from wild type to almost complete loss of function [17]. These mutations are excellent materials for understanding gene function, genetic regulation and compensatory processes [18]. Moreover, alleles generated by TILLING can be used in traditional breeding programs since the technology is non-transgenic and the mutations are stably inherited.

The main objectives of the present work were to investigate *Psy1* function and genetic regulation using three complementary reverse genetics approaches. *Psy1* was specifically silenced in wheat grain by RNAi to confirm *Psy1* function. Comparative analysis of transcriptome data between transgenic lines and non-transformed controls by RNA-Seq was used to reveal the transcriptional regulation network responding to *Psy1* down-regulation. In addition, two EMS (ethyl methanesulfonate)-mutagenised wheat populations were screened for mutations in *Psy1* by TILLING to obtain a series of *Psy1* alleles with potential to increase our understanding of the gene function, genetic regulation and compensatory processes. This integrative approach provided new insights into the molecular basis and regulatory processes of carotenoid biosynthesis in wheat grain.

## Methods

### Wheat transformation and regeneration

**Fig. 1:**
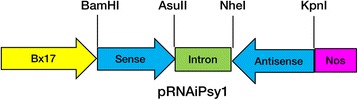
Non-scale diagram of the RNAi cassette in the transformation plasmid pRNAiPsy1. The trigger fragment of *Psy1* was placed in forward (Sense) and reverse (Antisene) orientations separated by the fourth intron of the wheat *Psy1* gene (Spacer). Restriction sites used in the RNAi vector construction are indicated. Bx17, endosperm-specific promoter; Nos, *Agrobacterium tumefaciens* nopaline synthase (*Nos*) terminator

The binary vector pSAABx17 containing the endosperm-specific promoter of HMW-GS (High-Molecular-Weight Glutenin Subunits) *Bx17*, the nopaline synthase (*Nos*) terminator, and a selectable neomycin phosphotransferase II (*npt II*) gene, was used to construct an RNAi vector. The first exon of *Psy1* (EF600063; 460 bp) was selected as the trigger fragment. Briefly, the sense fragment of *Psy1* was amplified using the primer pair PS-F containing a *Bam*HI site and PS-R with an *Asu*II site, while the antisense fragment was amplified with primers PA-F containing a *Kpn*I site and PA-R including a *Nhe*I site (Additional file 1: Table S1). The fourth intron of *Psy1* as the spacer was amplified by primers In-F and In-R. All sequences and directions of the inserts were confirmed by sequencing. The final RNAi construct was named pRNAiPsy1 (Fig. 1).

pRNAiPsy1 was transformed into wheat cultivar NB1 by *Agrobacterium tumefaciens*-mediated transformation [19]. Briefly, immature seeds were collected at 14 DPA and sterilized with 70 % ethanol for 1 min, 20 % bleach for 15 min and rinsed three times with sterile water. Isolated immature embryos were precultured on the induction medium for 4 d in dark at 25 °C. Then, the embryos were inoculated with a drop of *A. tumefaciens* suspension and co-cultured for 3 d on the same medium. The immature embryos were cultured on selection medium at 25 °C in the dark for 3 weeks for callus induction. Then, the calli were transferred onto regeneration medium at 25 °C in the light with a density of 45 μmol m^−2^ s^−1^ and 16 h photoperiod for another 3 weeks for differentiation process. The culture media are shown in Additional file 2: Table S2. All materials used for RNAi were kept at Crop Research Institute, Shandong Academy of Agricultural Sciences.

Regenerated plants were screened using G418. Surviving plants were transferred to soil and grown to maturity under growth chamber conditions of 22/16 °C day/night temperatures, 50–70 % relative humidity, 16 h photoperiod, and light intensity of 300 μmol photons m^−2^ s^−1^. Transformed plants were verified by PCR using specific primer pairs designed for the *FAD2* intron, a part of the pSAABx17 vector (Additional file 1: Table S1). Positive transgenic plants were self-pollinated and harvested in the following generations. T_3_ transgenic lines and non-transformed controls were grown under field conditions in Jinan, Shandong province, during the 2013–14 cropping season. Seeds were sown in 2 m rows with 20 plants per row, 30 cm between rows and 3 rows per transgenic line. Transformed plants were verified by PCR and tagged at anthesis. Grains for *Psy1* expression analysis were collected at 7-day intervals from 7 to 28 days post anthesis (DPA), immediately frozen in liquid nitrogen, and stored at −80 °C. Mature grains were harvested for YPC assays.

### RNA extraction and gene expression analysis

Total RNA was extracted from grains of T_3_ transgenic lines and non-transformed controls at different developmental stages using an RNAprep Pure Plant Kit (Tiangen Biotech, Beijing, China), and then treated with DNase I (Qiagen, Valencia, CA, USA), according to the manufacturer’s instructions. RNA purity and concentration were measured using a NanoDrop-2000 spectrophotometer (Thermo Scientific, Wilmington, DE, USA). RNA integrity was evaluated on agarose gels. Reverse transcription was performed with 1 μg of total RNA using a PrimeScript™ RT Reagent Kit (Takara Bio Inc., Otsu, Japan) following the manufacturer’s recommended protocol.

Quantitative real-time PCR (qRT-PCR) was performed on a Roche LightCycler 480 (Roche Applied Science, Indianapolis, IN, USA) in 20 μl reaction mixtures containing 10 μl of LightCycler FastStart DNA Master SYBR Green (Roche Applied Sciences), 0.4 μM of each primer, 50 ng of cDNA and 8.2 μl of ddH_2_O. Amplification conditions were an initial 95 °C for 10 min, and 40 cycles of 95 °C for 15 s, 60 °C for 20 s and 72 °C for 20 s. Fluorescence was acquired at 60 °C. Designs for gene-specific primer amplifying all three *Psy1* genes were based on conserved regions among the A, B and D subgenomes. Expression of a *β-actin* gene was used as an endogenous control to normalize expression levels of different samples. The primers are listed in Additional file 3: Table S3. Specificities of primers were confirmed by sequencing qRT-PCR products and melt curve analyses. Gene expression levels were presented as multiples of actin levels calculated by the formula 2^-ΔCT^ [ΔCT = (Ct value of target gene) − (Ct value of actin)] to correct for differential cDNA concentrations among samples [20]. For each line, three biological replicates, each with three technical replicates, were performed and the data were expressed as means ± standard error (SE).

### Yellow pigment content (YPC) assay

Grains from individual plants of T_3_ transgenic lines and non-transformed controls were ground into whole-grain flour by a Cyclotec™ 1093 mill (Foss Tecator Co., Hillerod, Denmark). The whole-grain flour (0.5 g) was used for YPC assay following Zhai et al. [21]. Three biological repeats were performed for each line, and each sample was assayed in duplicate; all differences between two repeats were less than 10 %.

### Transcriptome library construction and RNA sequencing

**Fig. 2:**
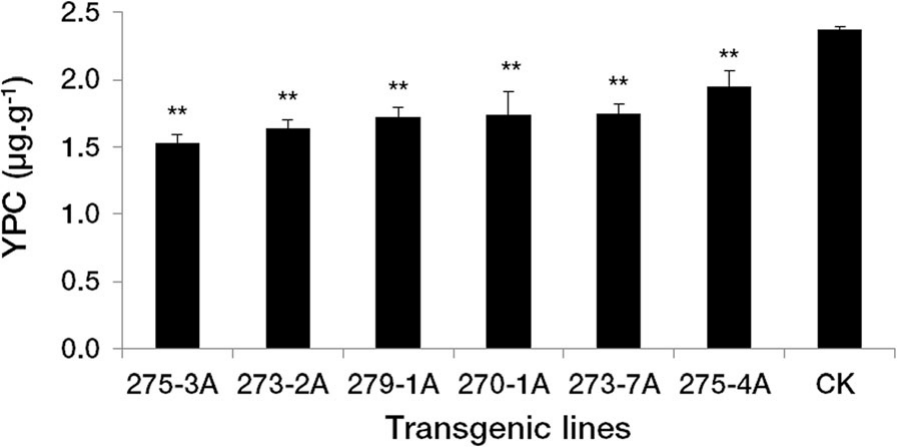
Yellow pigment content in grains from T_3_ transgenic lines and non-transformed controls. Data are presented as means ± standard error from three biological replicates. The double asterisks indicate significant differences between transgenic lines and controls at *P* = 0.01. CK, non-transformed controls

**Fig. 3:**
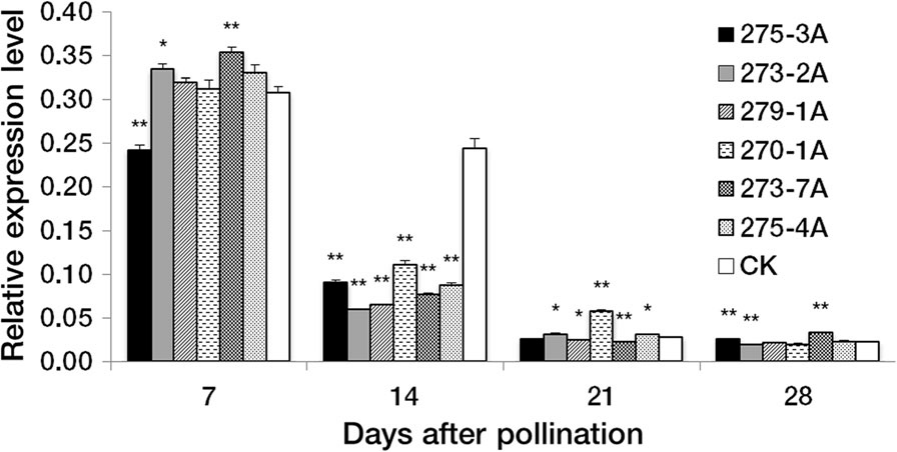
Expression levels of *Psy1* in developing grains from T_3_ transgenic lines and non-transformed controls. Gene expression levels were measured by qRT-PCR and normalized to the transcript level of a constitutively expressed *β-actin* gene in the same sample. Data are presented as means ± standard error from three biological replicates with three technical replicates each. Significant differences (Student’s *t* test) in transgenic lines compared to the controls are represented by one or two asterisks: * *P* <0.05, ** P <0.01. CK, non-transformed controls

To investigate the complex transcriptional regulation network underlying *Psy1* down-regulation, deep-sequencing analysis of transcriptomes of transgenic lines and non-transformed controls was performed by RNA-Seq. Three transgenic lines (275-3A, 273-2A and 279-1A) with the most significantly reduced YPC were selected (Fig. 2). Grains of transgenic lines and controls at 14 DPA were used for transcriptome analysis, because this developmental stage showed substantially decreased *Psy1* expression (Fig. 3). Total RNA were extracted from pooled grains of six biological repeats per transgenic line or controls and sent to BGI (Beijing Genomics Institute, Shenzhen, China) for RNA-Seq. Transcriptome libraries were prepared and sequenced on the Illumina HiSeq™ 2000 platform (Illumina, San Diego, CA, USA) following Zhou et al. [22].

### Screening and analysis of differentially expressed genes (DEGs)

Original image data were transformed into sequence data by base calling, and defined as raw reads. Before data analysis, it was prerequisite to remove dirty raw reads including reads with adaptors, those with more than 10 % of unknown bases and low quality reads (more than 50 % low quality bases). Clean reads were then aligned to the reference genome of *T. aestivum* (ftp://ftp.ensemblgenomes.org/pub/plants/release-26/fasta/triticum_aestivum/). Briefly, the clean reads were mapped to the genome reference by BWA software [23] and to the gene reference with Bowtie software [24]. Reads mapping to unique sequences, designated as unigenes, were the most critical subset in the transcriptome libraries as they explicitly identify a transcript. Unigene function was annotated by alignment of the unigenes with the NCBI (National Center for Biotechnology Information) non-redundant (Nr) database using Blastx at an E-value threshold of 10^−5^.

Gene expression level was normalized as the FPKM (fragments per kb per million reads) by a RSEM software package [25]. The fold-change in expression of each gene between the transgenic line and non-transformed control was evaluated by FPKM ratio. We used a false discovery rate (FDR) of <0.001 and the absolute value of |log_2_Ratio| ≥1 as the threshold to judge the DEGs. To obtain robust and reliable effects of *Psy1* down-regulation on gene transcription, only DEGs consistent across all three transgenic lines were chosen for subsequent analysis. Gene ontology (GO) annotation was conducted using the Blast2GO program (https://www.blast2go.com/). The GO categorizations were displayed as three hierarchies, namely biological process (BP), cellular component (CC) and molecular function (MF) by WEGO software [26]. DEGs were also analyzed against the KEGG database (Kyoto Encyclopedia of Genes and Genomes; http://www.genome.jp/kegg/) to explore the potential metabolic pathways that might be involved in reduction of carotenoid synthesis in transgenic lines.

### Subcellular localization of PSY1 in wheat

To investigate subcellular localization of PSY1, the cDNA sequence of *Psy1* without the termination codon was isolated from common wheat cultivar Jimai 22 (developed by the Crop Research Institute, Shandong Academy of Agricultural Sciences) using primers, Psy1-GFP-F (5′-GCCCAGATCAACTAGTATGGCCACCACCGTCACGCTGC-3′) and Psy1-GFP-R (5′-TCGAGACGTCTCTAGAGGTCTGGTTATTTCTCAGTG-3′), and confirmed by sequencing. The cDNA of *Psy1* was then C-terminally fused to the green fluorescent protein (GFP) gene in the pAN580 vector to create Psy1-GFP under the control of the cauliflower mosaic virus (CaMV) 35S promoter. The Psy1-GFP fusion and GFP were transiently transformed into wheat protoplasts following Zhang et al. [27]. Briefly, the stem and sheath of 30 wheat seedlings were cut into approximately 0.5 mm strips, which were immediately transferred into 0.6 M mannitol for 10 min in the dark. After discarding the mannitol, the strips were incubated in an enzyme solution for 4–5 h in the dark with gentle shaking (60–80 rpm). Then, an equal volume of W5 solution was added, followed by vigorous shaking by hand for 10 s. Protoplasts were released by filtering through 40 μm nylon meshes into round bottom tubes with 3–5 washes of the strips using W5 solution. The pellets were collected by centrifugation at 1,500 rpm for 3 min, and were then resuspended in MMG solution. Then, PEG-mediated transfections were carried out [28]. Fluorescence images were observed by a Zeiss LSM710 confocal laser microscope (Carl Zeiss MicroImaging GmbH, Germany).

### EMS mutagenesis

Two EMS-mutagenised common wheat populations were constructed following Slade et al. [17] with minor modifications. In brief, approximately 5,000 seeds of common wheat cultivars Jimai 22 and Jimai 20 (developed by the Crop Research Institute, Shandong Academy of Agricultural Sciences) were treated overnight with 1.2 % EMS solution and surviving plants were grown to maturity. Seeds from the leading spikes of the M_1_ plants were harvested and one grain from each plant was sown to generate the M_2_ population (Jimai 20: 1,250 lines; Jimai 22: 1,240 lines). Genomic DNA was isolated from individual M_2_ plants for TILLING analysis. Twenty seeds from each M_2_ line containing a mutation in the *Psy1* gene and wild type were grown under field conditions for further analysis.

### Mutation screening by TILLING

DNA samples were extracted from individual M_2_ plants of EMS-mutagenised populations derived from Jimai 20 and Jimai 22. DNA concentration was measured by a NanoDrop-2000 spectrophotometer (Thermo Scientific) and standardized. Equal amounts of DNA from individual plant samples were pooled eightfold and organized into 96-well plates. The optimal target region for TILLING screening, considered as one of the most promising for identifying mutations affecting protein function, was defined by the program CODDLE (Codons Optimized to Discover Deleterious Lesions; http://blocks.fhcrc.org/proweb/coddle/). In conjunction with the CODDLE results, homoeolog-specific primers were designed taking advantage of polymorphisms among the three homoeologs of *Psy1* in the hexaploid genome (Additional file 4: Table S4). Primer specificities were validated using Chinese Spring nulli-tetrasomic lines and by sequencing.

A fast and cost-effective method, mismatch-specific endonuclease digestion of heteroduplexes followed by non-denaturing polyacrylamide gels stained with silver, was used for mutation detection, which has similar sensitivity to traditional LI-COR screens [29]. Once a positive individual was found, the amplified product was sequenced to determine the accuracy of the mutation.

PARSESNP (Project Aligned Related Sequences and Evaluate SNPs; http://blocks.fhcrc.org/proweb/parsesnp/) was used to indicate the nature of each mutation. The PARSESNP and SIFT (Sorting Intolerant from Tolerant; http://sift.bii.a-star.edu.sg/) programs were used to predict the severity of each mutation. Mutations are predicted to have a severe effect on protein function if PSSM scores are >10 and SIFT scores are <0.05 [30, 31].

### Creation and characterization of F_2_ populations

To determine the impact of new *Psy1* alleles on protein function, homozygous M_3_ mutants carrying non-silent (including truncation and missense) mutations were backcrossed to corresponding wild type plants (Jimai 20 or Jimai 22) to reduce background noise. F_1_ plants were self-pollinated and harvested separately. Two hundred F_2_ seeds from each backcross and wild type were grown under field conditions in Beijing during the 2013–14 cropping season, arranged in a randomized complete block design. Seeds were sown in 2 m rows with 20 plants per row, 30 cm between rows and 10 rows per F_2_ population. Three genotypes (homozygous mutant, heterozygous mutant and wild-type genotype) in each F_2_ population were selected by sequencing. Spikes of five biological replicates for each genotype were tagged at anthesis. Immature grains were collected at 7-day intervals from 7 to 28 DPA for *Psy1* expression analysis. Mature grains were harvested for YPC assays. All F_2_ populations were conserved at the Crop Germplasm Resources Conservation Center, Chinese Academy of Agricultural Sciences.

The impacts of new *Psy1* alleles on YPC were assessed by comparing the differences between homozygous and heterozygous mutants with wild-type genotypes in each F_2_ population. YPC was measured by the method described above. All measurements were based on five biological repeats. Wild-type genotypes in each F_2_ population were designated as the calibrator with its value set to 1. The data are presented as means ± SE.

qRT-PCR was performed on cDNA from developing grains of each genotype in each F_2_ population at 7, 14, 21 and 28 DPA to investigate the effect of mutations on the expression pattern of the particular *Psy1* gene and its homoeologs. Briefly, total RNA was extracted from pooled grains of five biological repeats per genotype. Two sets of primers were designed by comparing coding regions of the three *Psy1* homoeologs. The first set of primers amplifying all three homoeologs was used to examine gene-specific expression. The second set, the homoeolog-specific primers, was used to determine expression levels of each homoeolog (Additional file 3: Table S3). The specificity of these primers was tested as described above. The protocol for qRT-PCR was also the same. For each sample three technical replicates were performed. Relative expression was calculated using the 2^-ΔΔCT^ method [20]. Relative expression levels of *Psy1* and its homoeologs were normalized firstly to the transcript level of *β-actin* gene in the same sample and then calculated relative to the value of wild-type genotypes at 28 DPA (set to 1) in each F_2_ population. Expression analysis was performed only on F_2_ populations for the mutants with significant phenotypic changes.

### Functional domains and structural modeling of wheat PSY1

Functional domains of PSY1 protein were predicted by the NCBI’s Conserved Domain Database (CDD; http://www.ncbi.nlm.nih.gov/Structure/cdd/cdd.shtml). To understand the effect of new *Psy1* alleles on protein structure, the three-dimensional structure of PSY1 was generated by the SWISS-MODEL (http://swissmodel.expasy.org/) and visualized using Swiss-PdbViewer (http://www.expasy.org/spdbv/).

### Detection of alternative splicing variants

Splice junction mutations are speculated to have severe effects on protein function because they can lead to aberrant RNA splicing and subsequently altered or truncated protein translation [32]. Although no splice junction mutation was identified in this study, mutation sites in M090122 and M092201 were adjacent to the splice site. The mutation site in M090122 was localized at the 3′ end of exon II and that in M092201 was at the second nucleotide from the 3′ end of exon V. Reverse transcription PCR was performed to investigate whether these mutations led to alternative splicing. Briefly, total RNA were extracted from homozygous mutant and wild type individuals, and reverse transcribed into cDNA by the method described above. The cDNA were amplified using the corresponding primers (Additional file 5: Table S5), and PCR products were analyzed by gel electrophoresis and sequenced.

### Statistical analysis

Data are presented as means ± SE. Student’s *t* test was used to assess the statistical significance of differences in pairwise comparisons of transgenic lines and non-transformed controls, or between homozygous or heterozygous mutants and wild-type genotypes in each F_2_ population.

## Results

### *Psy1* gene expression and YPC in grains of transgenic lines

The 460 bp trigger fragment from *Psy-A1* that was used for the RNAi vector construction shared 90 % and 95 % sequence similarity with *Psy-B1* and *Psy-D1*, respectively. Using the *Agrobacterium*-mediated transformation method six positive, non-segregating transgenic lines, designated as 275-3A, 273-2A, 279-1A, 270-1A, 273-7A and 275-4A, were obtained. They showed no differences in morphology and development compared to non-transformed controls.

The effect of the transformed *Psy1*-hairpin on *Psy1* expression was examined in six positive T_3_ transgenic lines during grain development. At 7 DPA, qRT-PCR analyses showed a significantly decreased transcript level of *Psy1* in the transgenic line 275-3A (*P* <0.01), significantly increased transcription levels in 273-2A and 273-7A (*P* <0.05 and *P* <0.01, respectively), and slight changes in the other lines, compared to non-transformed controls. Substantially decreased *Psy1* expression levels of 54–76 % were found in all transgenic lines at 14 DPA (*P* <0.01). At 21 and 28 DPA differences in expression levels between the transgenic lines and controls were very small (2–15 %), except for line 270-1A at 21 DPA and line 273-7A at 28 DPA (Fig. 3). Significantly decreased YPC ranging from 26 to 35 % occurred in all transgenic lines compared with non-transformed controls (Fig. 2).

### Transcriptional profiling underlying *Psy1* down-regulation

Totals of 1,128,107, 1,160,285, 1,192,915 and 1,228,928 unigenes were obtained for transgenic lines 273-2A, 275-3A, 279-1A and the control, respectively (Additional file 6: Table S6). Comparison of the transcript abundances between transgenic lines and controls identified 948, 930 and 992 DEGs for 273-2A, 275-3A and 279-1A, respectively (Additional file 6: Table S6). In total, 287 DEGs were consistent across all three transgenic lines, perhaps representing the reliable effects of *Psy1* down-regulation on gene transcription (Additional file 7: Table S7).

**Fig. 4:**
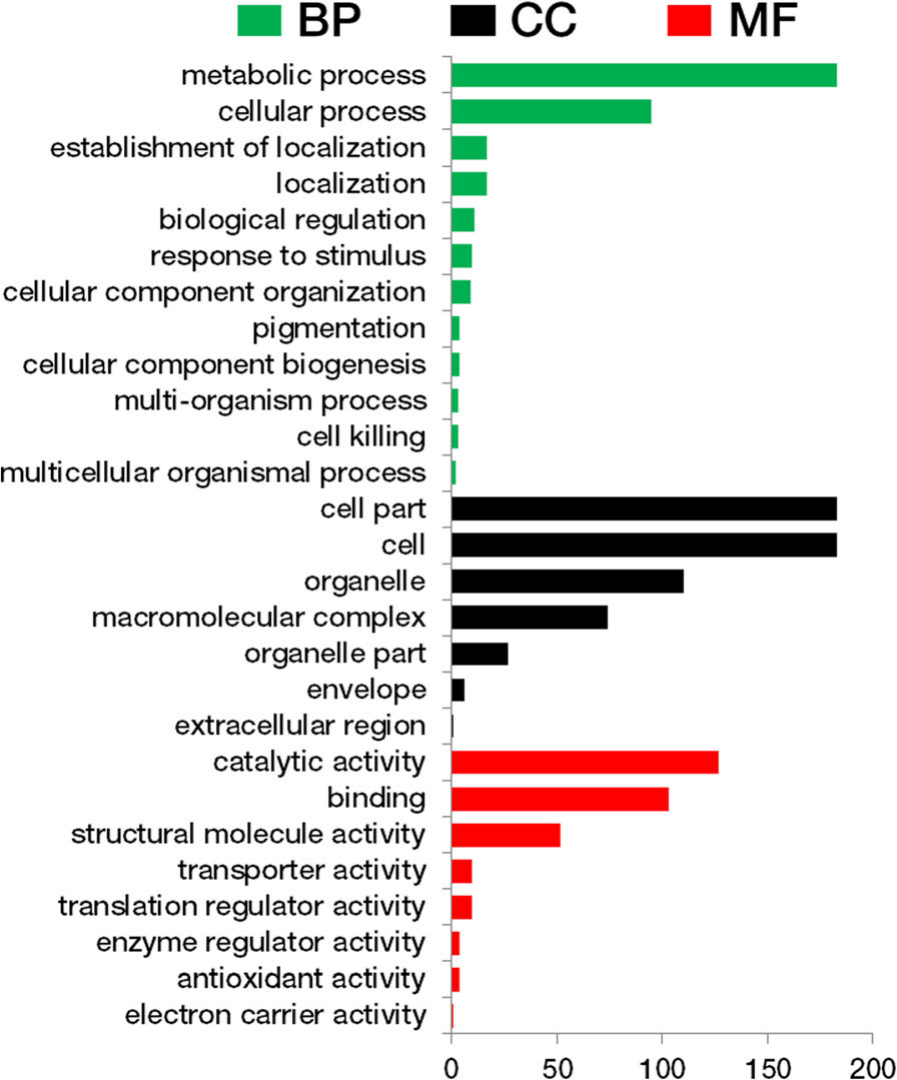
Gene ontology classifications of differentially expressed genes (DEGs) consistently present in all transgenic lines. Because a gene can be assigned to more than one GO term, the sum of genes in each category may exceed the number of DEGs (287). BP, Biological process; CC, Cell component; MF, Molecular function

**Fig. 5:**
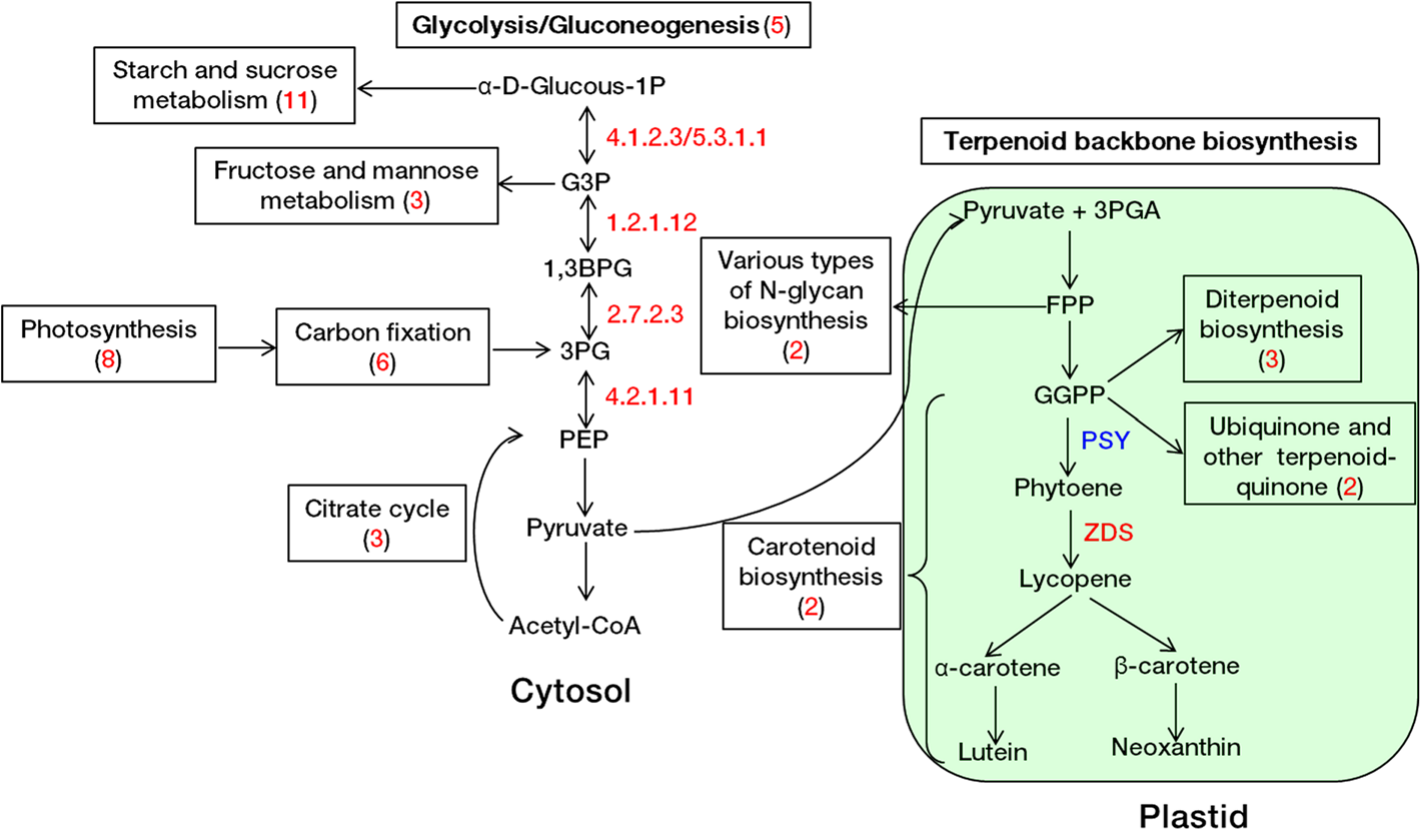
Overview of major metabolic pathways associated with *Psy1* down-regulation in transgenic lines. Genes that were 2-fold greater up- or down-regulated are shown in red or blue, respectively. The number of candidate genes in a relevant pathway is indicated in brackets, and the detail of candidate genes in each pathway is listed in Table S7. 1,3BPG, 3-phospho-D-glyceroyl phosphate; 3PG, 3-phospho-D-glycerate; FPP, farnesyl diphosphate; G3P, glyceraldehyde 3-phosphate; GGPP, geranylgeranyl pyrophosphate; PEP, phosphoenolpyruvate; PSY, phytoene synthase; ZDS, zeta-carotene desaturase

Categorization of GO terms of the 287 DEGs is shown in Fig. 4. Metabolic process and cellular process were the major categories annotated to the biological process (BP); cell part and cell were the major categories annotated to the cellular component (CC); and catalytic activity and binding were the major categories annotated to the molecular function (MF). Through pathway enrichment analysis, 199 of the 287 DEGs were assigned to 46 metabolic pathways (data not shown). The pathways significantly associated with *Psy1* down-regulation included carotenoid biosynthesis, diterpenoid biosynthesis, various types of N-glycan biosynthesis, ubiquinone and other terpenoid-quinone, glycolysis/gluconeogenesis, starch and sucrose metabolism, fructose and mannose metabolism and citrate cycle, photosynthesis, and carbon fixation in photosynthetic organisms (Fig. 5). All candidate genes in relevant pathways are listed in Additional file 8: Table S8.

### PSY1 subcellular localization

**Fig. 6:**
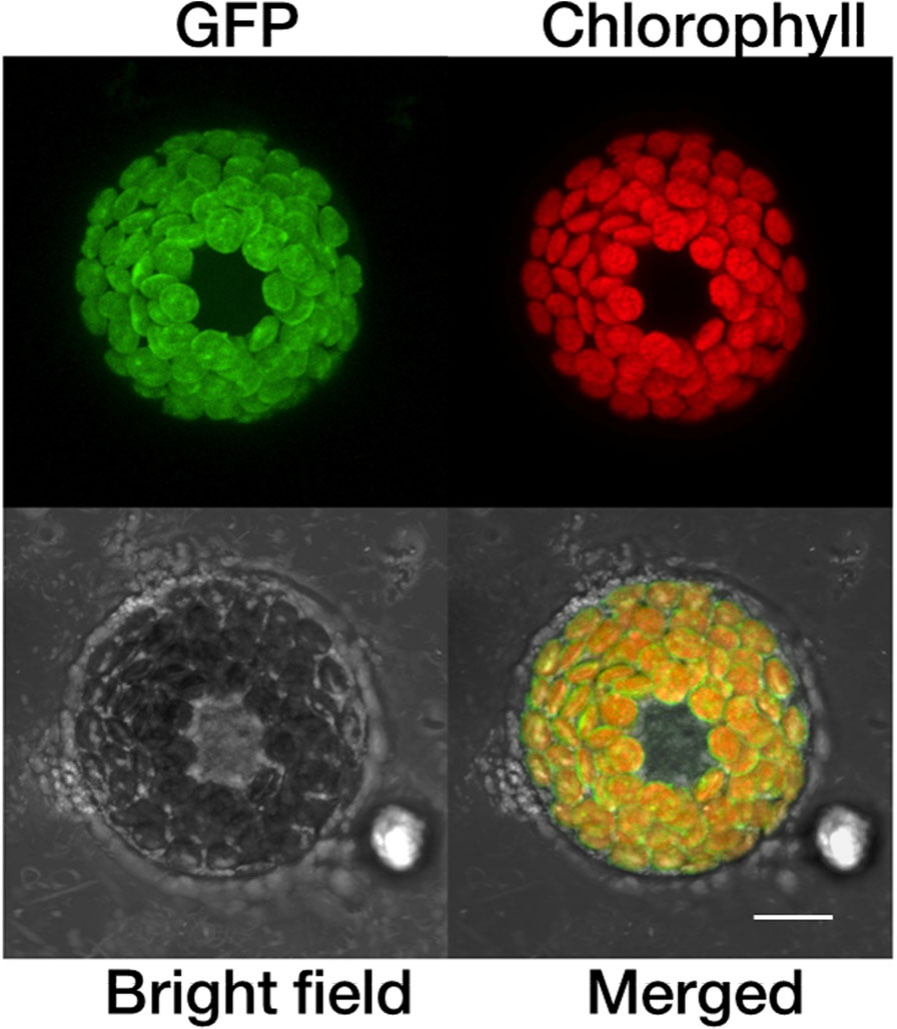
Subcellular localization of PSY1 in wheat protoplasts by confocal microscopy. GFP (green), chlorophyll autofluorescence (red), bright-field, and an overlay of green and red signals are shown. Bar, 10 μm

Psy1-GFP was constructed and transiently expressed in wheat protoplasts to investigate PSY1 subcellular localization. Protoplasts allow us to observe the localization of transiently expressed PSY1 proteins, due to retain their tissue specificity after isolation and thereby reflect in vivo conditions. GFP alone was distributed evenly in the cytoplasm and nuclei (data not shown), whereas the Psy1-GFP fusion proteins co-localized exclusively with autofluorescence signals of chlorophyll, indicating that PSY1 was localized in plastids (Fig. 6).

### Identification of mutations in *Psy1* by TILLING

Eighty two new *Psy1* alleles were identified in the two EMS-mutagenised populations, including three truncation, 26 missense and 53 silent mutations (Table 1; Additional file 9: Table S9). As expected for alkylation of guanine by EMS, the majority of mutations were G to A (61.0 %) or C to T (31.7 %) transitions, with the exception of six mutations as follows: A to C (2), A to G, A to T, T to C and T to G.

**Table 1:** Summary of non-silent mutations in *Psy1* identified by TILLING

Gene	M3 Plant	Cultivar	Exon\Intron	Nucleotide change^a^	Amino acid change^b^	Codon change	Zygosity^c^
*Psy-A1*	M091753	J22	Exon	C308T	A103V	GCA → GTA	Hom
	**M090158** ^d^	J20	Exon	C3201T	Q346*	CAG → TAG	Hom
	M092432	J20	Exon	C3255T	L364F	CTT → TTT	Hom
	M091887	J22	Exon	C335T	S112L	TCG → TTG	Hom
	**M091949**	J22	Exon	C349T	Q117*	CAG → TAG	Hom
	**M090950**	J22	Exon	G1224A	W172*	TGG → TAG	Hom
	**M091151**	J22	Exon	G1230A	R174K	AGG → AAG	Hom
	M090997	J22	Exon	G271A	E91K	GAG → AAG	Hom
	M092152	J22	Exon	G3231A	E356K	GAG → AAG	Het
	M091102	J22	Exon	G3554A	R397K	AGG → AAG	Hom
	M090333	J20	Exon	G3605A	G414E	GGG → GAG	Het
	M092889	J22	Exon	G371A	R124K	AGG → AAG	Hom
	M090755	J20	Exon	G400A	G134R	GGG → AGG	Hom
	M092383	J20	Exon	G412A	A138T	GCC → ACC	Het
	M091295	J22	Exon	G436A	E146K	GAG → AAG	Hom
	M092101	J22	Exon	G596A	E160K	GAG → AAG	Hom
	**M090122**	J20	Exon	G629A	V171G	GTA → AGT	Hom
	M092853	J22	Exon	T3169G	V335G	GTC → GGC	Het
*Psy-B1*	M091983	J22	Exon	G2073A	E244K	GAG → AAG	Het
*Psy-D1*	M092201	J20	Exon	C3792T	P370L	CCG → CTG	Het
	M091755	J22	Exon	C4109T	P409S	CCT → TCT	Hom
	M090628	J20	Exon	C4110T	P409L	CCT → CTT	Hom
	M091169	J22	Exon	G1347A	D217N	GAC → AAC	Het
	**M091217**	J22	Exon	G3609A	R309K	AGA → AAA	Hom
	M090649	J20	Exon	G3761A	V360M	GTG → ATG	Hom
	M090324	J20	Exon	G3779A	E366K	GAG → AAG	Het
	M091365	J22	Exon	G4049A	D389N	GAC → AAC	Het
	M092126	J22	Exon	G4071A	R396K	AGG → AAG	Hom
	M090608	J20	Exon	G4097A	V405M	GTG → ATG	Het

^a^the first letter indicates the wild type nucleotide, the number is its position from the start codon, and the last letter is the mutant nucleotide

^b^the first letter indicates the wild type amino acid, the number is its position from the smethionine, and the last letter is the mutant amino acid

^c^Hom, homozygous genotype; Het, heterozygous genotype

^d^bold items, mutations severely affecting phenotype

^e^*, termination mutation

Two missense mutations (M090628 and M091151) and three truncation mutations (M090158, M090950 and M091949) were predicted to have severe effects on protein function based on SIFT score and PSSM values (Table 2).

**Table 2:** Mutations severely affecting protein function as predicted by the PARSESNP and SIFT programs^a^

Gene	Mutant	Cultivar	Nucleotide change^b^	Amino acid change^c^	PSSM	SIFT
*Psy-A1*	M091151	J22	G1230A	R174K	16.2	0.03
*Psy-D1*	M090628	J20	C4110T	P409L	18	0.04
*Psy-A1*	M090158	J20	C3201T	Q346*		
*Psy-A1*	M090950	J22	G1224A	W172*		
*Psy-A1*	M091949	J22	C349T	Q117*		

^a^High PSSM (>10) and low SIFT scores (<0.05) predict mutations with severe effects on protein function. PSSM and SIFT scores are not reported for mutations that produce premature termination codons

^b^The first letter indicates the wild type nucleotide, the number is the position from the start codon, and the last letter is the mutant nucleotide

^c^The first letter indicates the wild type amino acid, the number is the position from the methionine, and the last letter is the mutant amino acid

### Characterization of new alleles of *Psy1*

**Fig. 7:**
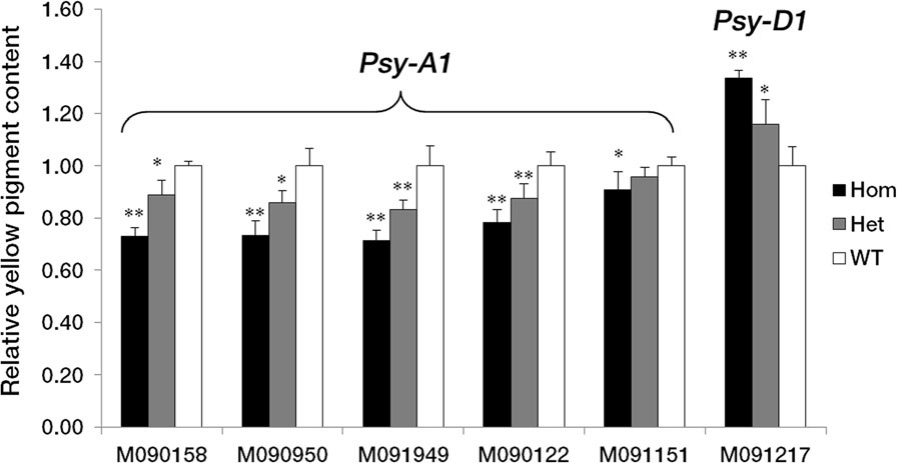
Relative yellow pigment content of different mutant genotypes in F_2_ populations. F_2_ populations were derived from homozygous non-silent (truncation and missense) mutants crossed with corresponding controls (Jimai 20 or Jimai 22). Data are given as fold measures relative to wild-type genotypes in each F_2_ population (set to 1). Five biological replicates were performed for each comparison and the data are presented as means ± standard error. Significant differences (Student’s *t* test) between homozygotes and heterozygotes for the presence of the mutation and wild-type genotypes in each F_2_ population are represented by one or two asterisks: * *P* <0.05, ** P <0.01. Hom, homozygous mutants; Het, heterozygous mutants; WT, wild-type genotypes

Twenty-nine F_2_ populations were developed from homozygous M_3_ mutants carrying non-silent (missense and truncation) mutations and corresponding wild type plants, and YPC assays were carried out to characterize the effects of the non-silent mutations on protein function. As shown in Fig. 7 mutations in *Psy-A1*, namely M090158, M090950, M091949, M090122 and M091151, significantly reduced YPC by 9–29 % (between homozygous mutants and wild-type sibs), whereas the mutation in *Psy-D1* of M091217 significantly increased YPC by 34 %.

**Fig. 8:**
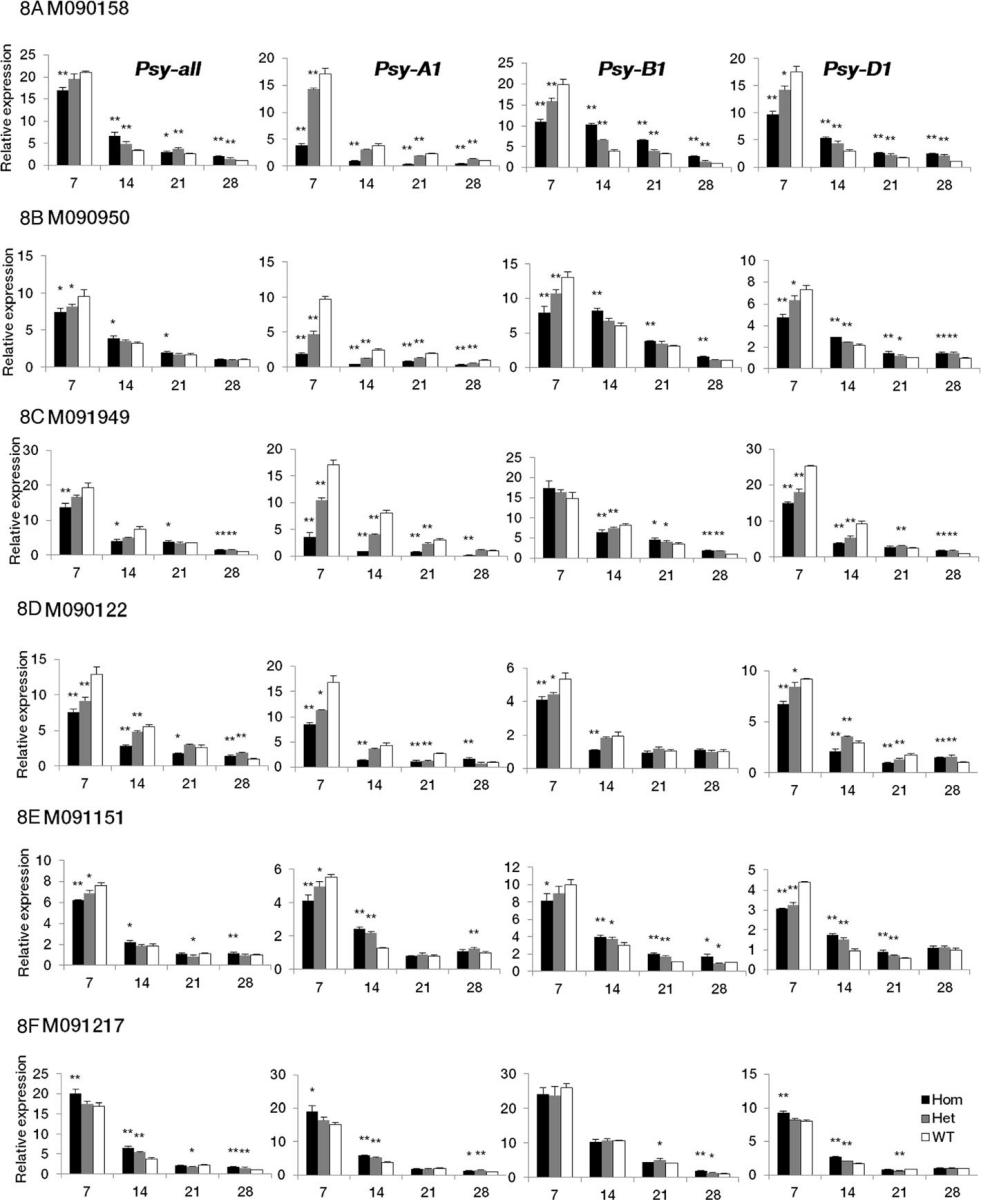
Expression analysis of *Psy1* and its homoeologs in developing grains of three genotypes in each F_2_ population. **a** M090158. **b** M090950. **c** M091949. **d** M090122. **e** M091151. **f** M01217. For each genotype, five biological repeats were sampled and pooled for RNA extraction and gene expression analysis. Transcript levels are given as expression levels relative to the values of wild-type genotypes at 28 DPA (set to 1) after normalization to *β-actin* level. Data are presented as means ± standard error from three technical replicates. Significant differences (Student’s *t* test) between homozygous and heterozygous mutant individuals and wild-type genotypes in each F_2_ population are represented by one or two asterisks: * *P* <0.05, ** P <0.01. Hom, homozygous mutants; Het, heterozygous mutants; WT, wild-type genotypes

The expression profiles of *Psy1* and its homoeologs in grains of each genotype in the six F_2_ populations were determined by qRT-PCR at 7, 14, 21 and 28 DPA (Fig. 8). In three populations derived from truncation mutations in *Psy-A1* (M090158, M090950 and M091949), *Psy-A1* expression levels in homozygous mutants were reduced to 11–48 % compared to wild-type sibs during grain development. Compensatory responses from the B and D subgenomes were found to begin at 14 or 21 DPA. For two populations derived from missense mutations in *Psy-A1* (M091151 and M090122), the *Psy-A1* expression levels in homozygous mutants were more than 33 % of that in wild-type plants, and the compensatory response began at 14 or 28 DPA. For the population derived from the missense mutation in *Psy-D1* of M091217, the expression profiles of *Psy1* and its homoeologs in homozygous mutants were significantly higher than that of wild-type genotypes during all grain development, except for 21 DPA.

**Fig. 9:**
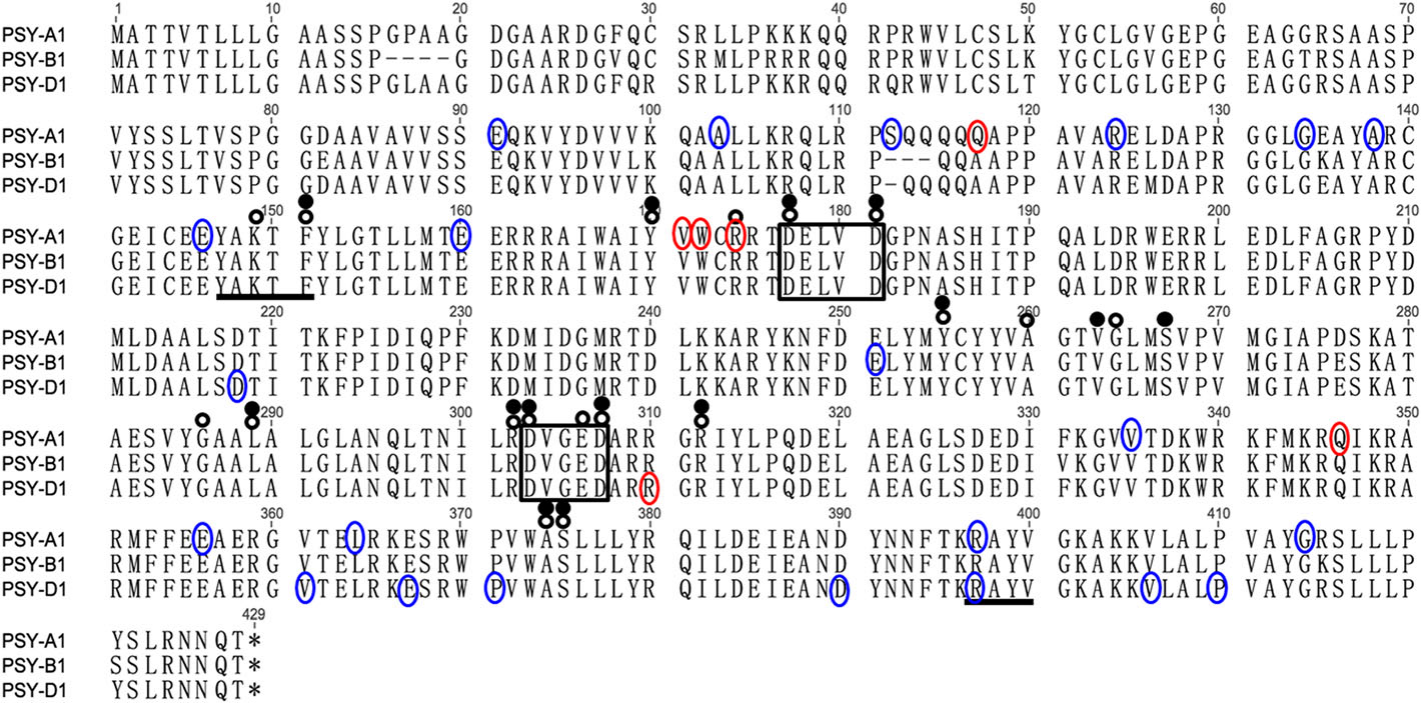
Functional domains of homoeologous PSY1 protein sequences. Amino acid sequences of PSY1 were analyzed using the NCBI’s Conserved Domain Database. Numbers above the alignment indicate the amino acid positions along the PSY-A1 protein. Framed, aspartate rich regions (DXXXD; substrate-Mg^2+^-binding sites); open black circle, substrate binding pocket; filled circle, catalytic residues; line, active site lid residues; blue circle, missense mutations; red circle, mutations resulting in significant yellow pigment content change, including truncation and missense mutations

**Fig. 10:**
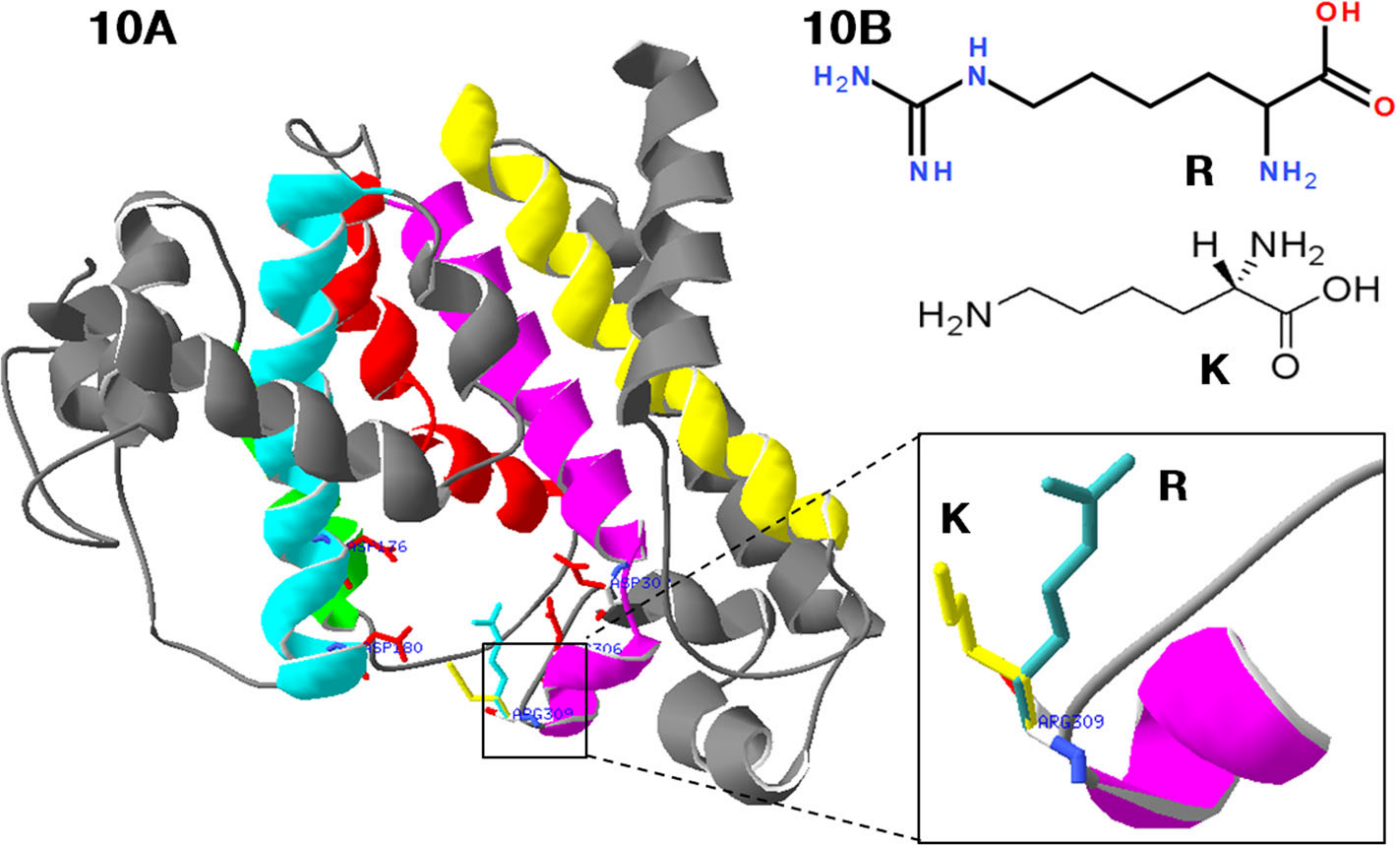
Graphical representation of PSY-D1 modeled by SWISS-MODEL. **a** Model of M091217 (R309K) superimposed with wild type. **b** Carbon skeleton of arginine (R) and lysine (K). The alpha helices at the locations of the substrate binding pocket and catalytic site are shown in bright colors (blue, red, yellow and purple); other helices are in grey. The carbon chain of conserved aspartate in aspartate rich regions (DXXXD) are shown in red, and the carbon chains of R and K are in blue and yellow, respectively

Based on the NCBI’s CDD, four characteristic domains were identified in PSY1 protein including aspartate rich regions (DXXXD; substrate-Mg^2+^-binding sites), a substrate binding pocket, catalytic residues, and active site lid residues (Fig. 9). For three missense mutations significantly influencing YPC and gene expression, the mutation sites of M090122 (V171I) and M091151 (R174K) were adjacent to the ^177^DXXXD^181^ domain, and the mutation in M091217 (R309K) was close to the ^302^DXXXD^306^ domain. Three-dimensional structure analysis showed that the mutation site of M091217 was located at the entrance of the substrate binding pocket in the PSY-D1 protein (Fig. 10).

### Alternative splicing

**Fig. 11:**
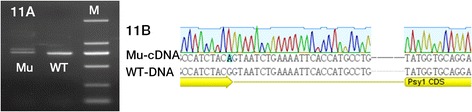
Alternative splicing in the M090122 mutant. **a** Reverse transcription PCR analysis showing alternative splicing in M090122. **b** Alignment of cDNA from homozygous M090122 mutant and DNA sequence of wild type. The sequence traces indicate that the G629A mutation in M090122 caused an alternative splice junction site, located 25 nucleotides downstream of the normal splice junction. Mu, mutagenised line; WT, wild type; M, molecular weight standard DL2000

The cDNA of grains from homozygous mutants M090122 and M092201 and wild type were amplified and sequenced to investigate the impact of the mutations on pre-mRNA splicing. PCR results for M090122 revealed two products of different size, compared to only the smaller one in wild type individuals (Fig. 11). Sequences of the two transcripts showed that the larger product included a 25 bp fragment of intron II, that resulted in a frame-shift mutation causing a premature termination codon at position 226 (data not shown); the smaller fragment was the constitutive transcript. The M092201 mutant did not produce alternative splicing compared to wild type.

## Discussion

### *Psy1*-specific silencing

RNAi is a sequence-specific gene suppression system. Previous studies indicated that nucleotide identity between the trigger fragment and target gene is crucial for successful gene silencing by RNAi [33]. It has been suggested that effective gene silencing in higher plants requires 88–100 % nucleotide identity, and 81 % or less nucleotide identities are generally not sufficient for inducing strong and specific gene silencing [34]. In addition, the presence of a continuous stretch of similarity covering at least 21 identical nucleotides between the trigger fragment and target gene is required, although it may not always be sufficient for efficient gene silencing [35, 36]. In this study, the first exon of *Psy-A1* (460 bp) was selected as the trigger fragment; it shares 90 % and 95 % nucleotide identity with *Psy-B1* and *Psy-D1*, respectively. Additionally, there were also six contiguous stretches of identical nucleotides longer than 21 nt. As expected, all three *Psy1* homoeologs were simultaneously silenced, which was proven by RNA-seq (Additional file 7: Table S7).

In grasses, PSY are encoded by three paralogous genes (*Psy1-3*). The *Psy1*, *Psy2* and *Psy3* genes were located to the group 7, 5 and 5 chromosomes, respectively [37]. To determine the gene specificity of our RNAi construct, the sequence similarities among these three genes were analyzed. *Psy3* shared 75.4 % nucleotide identity with *Psy1* within the 460 bp trigger fragment and had no contiguous stretches of identical nucleotides over 16 nt. The sequence of the target region in *Psy2* was not obtained, but the nucleotide identity in the known region was only 74.4 % compared with *Psy1* (data not shown). Therefore, we inferred that the RNAi construct used in the study specifically silenced *P*s*y1* expression rather than *Psy2* and *Psy3*. In contrast to *Psy1*, the RNA-seq revealed that the expression levels of *Psy2* and *Psy3* were not significantly different between transgenic lines and controls (data not shown).

*Psy1* expression was not significantly reduced in most transgenic lines at 7 DPA, (Fig. 3), because the *Bx17* hardly expresses at this stage [38]. In contrast, *Psy1* expression level was substantially decreased in all transgenic lines at 14 DPA; this might be attributed to the highest expression level of *Bx17* and higher expression of *Psy1*. In the later developmental stages, the *Bx17* expression was still very high, whereas *Psy1* expression was not reduced distinctly in transgenic lines compared to controls, due to the low expression level of *Psy1* and the basic demand of carotenoids for normal growth of plants.

### The effect of *Psy1* down-regulation

Quantitative timing analysis of *Psy1* expression showed that the RNAi effect was the greatest at 14 DPA, generating 54–76 % reductions compared to non-transformed controls. As expected, all transgenic lines showed significant YPC reductions, confirming the importance of *Psy1* for carotenoid accumulation in wheat grains.

In general, plants have the flexibility to cope with enhancements or reductions of gene products by coordinating the transcriptional regulation network. Pleiotropic effects correlated with up- or down-regulation of *Psy* genes were reported previously [39], indicating a strong correlation between carotenoid biosynthesis and core metabolism, such as photosynthesis, starch and sucrose metabolism, glycolysis/gluconeogenesis, and the citrate cycle [40–42]. In this study, some candidate genes involved in secondary metabolic pathways and core metabolic processes were found to collectively participate in the adaptive process of *Psy1* down-regulation based on RNA-Seq analysis (Fig. 5; Additional file 8: Table S8). In the carotenoid pathway, except for *Psy1* down-regulation, up-regulation of the zeta-carotene desaturase gene (*Zds*) might be attributed to feedback from reduction of downstream products. Some genes involved in various types of N-glycan biosynthesis, ubiquinone and other terpenoid-quinone biosynthesis and diterpenoid biosynthesis, were up-regulated in transgenic lines. These secondary metabolic pathways compete for FPP (farnesyl diphosphate) or GGPP (geranylgeranyl pyrophosphate) with carotenoid biosynthesis, and therefore carotenoid biosynthesis reduction induces more precursors flow into other pathways. Genes coding enolase (EC 4.2.1.11), phosphoglycerate kinase (EC 2.7.2.3), glyceraldehyde 3-phosphate dehydrogenase (EC 1.2.1.12), fructose-bisphosphate aldolase (EC 4.1.2.13) and triosephosphate isomerase (EC 5.3.1.1) were up-regulated, which might favor the flow into gluconeogenesis since transgenic lines needed a lower flux through and out of the glycolytic pathway for carotenoid biosynthesis. Enhancement of storage reserves synthesis, such as fructose and mannose metabolism and starch and sucrose metabolism, also proved this point. Additionally, enhanced gluconeogenesis further induced photosynthesis, carbon fixation in photosynthetic organisms and citrate cycle. These previously unrecognized YPC-related-genes in core metabolism established a broader basis for the molecular regulating carotenoid biosynthesis in wheat grains.

### Dissection of *Psy1* by TILLING

TILLING is a flexible strategy for exploring gene function and regulation, producing large series of mutated alleles that may affect protein function and generate partial phenotypic changes or intermediate expression of target genes. In this study, 29 non-silent (truncation and missense) mutations in *Psy1* genes in common wheat were identified, providing a resource not only for functional analysis, but also for understanding the importance of different amino acids and regions regulating the protein function, as well as to study compensatory responses.

The severity of each non-silent mutation was predicted by PARSESNP and SIFT, and YPC in each F_2_ population was measured. However, severity prediction was not always consistent with changes in phenotype. For example, the mutation in M090628 was predicted to have a severe effect on protein function, whereas it showed no significant phenotypic change. This might indicate that the conserved sequence had no direct role in controlling enzyme activity, since PARSESNP and SIFT do not account for active or conserved domains, but make predictions based on amino acid conservation and properties after an alignment search in the protein sequence database [30, 31].

Compared with missense mutations in *Psy-A1*, three truncation mutations showed stronger effects on *Psy-A1* expression by reducing *Psy-A1* transcript levels in homozygous mutants to 11–48 % of that in wild-type genotypes during whole grain development (Fig. 8). These reductions might be due to a quality control mechanism preventing accumulation of non-functional or deleterious truncated proteins, known as Nonsense Mediated mRNA Decay [43]. In wheat, significantly reduced RNA levels have also been reported for multiple genes containing premature termination codon mutations such as HMW glutenin subunit [44], waxy gene [45], and polyphenol oxidase gene [46].

TILLING is an efficient method to identify mutations in genes of interest, but the mutant effect is often masked by the presence of multiple copies of the same genes in polyploids, such as common wheat. In this study, the expression levels of three homoeologs were measured to study compensatory processes. Unexpectedly, the expression of all three *Psy1* homoeologs was significantly reduced or increased together at 7 DPA, except for *Psy-B1* in M091949 and M091217 (Fig. 8). In three truncation mutants, the compensatory responses from B and D homoelogs started at 14 DPA for M090158 and M090950 and at 21 DPA for M091949. For missense mutations in M091151 and M090122, the compensatory response began at 14 and 28 DPA, respectively. One possible reason for these phenomena was that the expression of all three *Psy1* homoeologs is coordinately regulated under normal conditions, but separately regulated under stress. Furthermore, we inferred that 14 DPA was an important stage for *Psy1* expression regulation during wheat grain development because most compensatory responses started at this stage. More detailed investigations are needed to substantiate these hypotheses. Compared with *Psy-B1*, the expression level of *Psy-A1* and *Psy-D1* showed more distinct changes, and it seems that they were more sensitive to expression regulation. RNA-seq data also showed that the order of down-regulation level among three homoeologs was *Psy-D1 > Psy-A1 > Psy-B1* in transgenic lines (Additional file 7: Table S7).

The nucleotide change (G3609A) in M091217 resulted in substitution of arginine by lysine at position 309 (R309K). The three-dimensional structure of PSY1 showed that this mutation was adjacent to the entrance of the substrate binding pocket in the PSY-D1 protein, and was possibly easier for substrate binding due to a shorter carbon chain (R to K) resulting in increased carotenoid accumulation (Fig. 10). This mutation might coordinately induce expression of all three *Psy1* homoeologs, although *Psy-B1* showed less changes (Fig. 8). Mutations in gene coding regions have potential to alter plant metabolism in ways other than changing the level of target gene products. For example, a mutated site may change the enzyme-substrate affinity, alter enzyme regulatory domains, or interfere with proper subunit or other protein-protein interactions. The aspartate rich region DXXXD is a conserved domain within isoprenoid synthases and forms an active site to bind phosphate groups of a substrate [47]. In this study, all missense mutants with severe effects on YPC were close to the DXXXD domain, indicating that these regions are very important for PSY1 function. Previous studies showed that sequence variations affecting the catalytic efficiency of the PSY enzyme were as subtle as a single amino acid [48]. Therefore, we infer that these mutations may affect the affinity of PSY1 for phosphate groups of a substrate and further influence carotenoid accumulation.

### Alternative splicing

Sequencing analysis of cDNA indicated that the G629A mutation in M090122 caused an alternative splice junction site, located 25 nucleotides downstream of the normal splice junction (Fig. 11). This mechanism was previously reported in plants and explained by local scanning of the spliceosome to select the best intron splice site based on sequence context [49]. The mutation resulted in a frame shift and a premature termination codon at position 226. We assume that the alternative splicing in M090122 might decrease the content of functional PSY1 protein and further reduce carotenoid biosynthesis. Alternative splicing of *Psy1* regulating enzyme activity and carotenoid accumulation was also reported in wheat and *Hordeum chilense* [50, 51].

### Molecular breeding

Mutants identified by TILLING are not involved in genetic modification and can be introduced into breeding programs. The use of mutagenesis in plant breeding is generally considered to have contributed to the release of more than 2,250 crop cultivars with improved yield and quality traits [52]. Therefore, mutants identified in this study will be useful as breeding germplasm for wheat quality improvement. For example, mutants M090158, M090950, M091949 and M090122 with significantly reduced YPC could be used in improvement of wheat genotypes for Chinese style foods such as steamed bread and white Chinese noodles where a bright whiteness is preferred. Meanwhile, M091217 with higher YPC could be useful for improving nutrition because carotenoids are important for human health. Furthermore, these mutants come from elite wheat cultivars Jimai 20 or Jimai 22 and are potentially useful without further pre-breeding to remove undesirable agronomic traits.

## Conclusion

The *Psy1* function and genetic regulation in common wheat were extensively analyzed using a complementary reverse genetics approach. The RNAi-mediated down-regulation of *Psy1* resulted in remarkable reduction in YPC, confirming the important impact of *Psy1* on carotenoid accumulation in wheat grains. Based on RNA-Seq and bioinformatics analysis, a series of candidate genes involved in both core metabolic processes and secondary metabolic pathways communicated and worked collaboratively to adapt to the *Psy1* down-regulation. The TILLING identified a suite of mutations in *Psy1* and provided a more in-depth insight into the gene function, genetic regulation, structure-function relationship, as well as the compensatory response. The aspartate rich region DXXXD, a conserved domain among isoprenoid synthases, was identified as an important region influencing PSY1 function in wheat, and conserved nucleotides adjacent to the domain influenced YPC by regulating gene expression, enzyme activity or alternative splicing. Moreover, the compensatory response played a vital role in gene expression during gain development and 14 DPA was considered as a key regulation node. The findings achieved in the present study would be helpful to further disclose the molecular basis and genetic regulation of carotenoid synthesis in wheat grains and could eventually facilitate the genetic improvement of wheat quality in the future.

## Additional files

Additional file 1: Table S1. Primers used for the RNAi vector construction and positive transgenic line detection. (DOCX 16.7 kb)
Additional file 2: Table S2. Culture media used in this study for callus induction and differentiation. (DOCX 16.7 kb)
Additional file 3: Table S3. Primers developed for qRT-PCR analysis. (DOCX 17.5 kb)
Additional file 4: Table S4. Homoeolog-specific primers developed for mutation detection by TILLING. (DOCX 17.9 kb)
Additional file 5: Table S5. Primers designed for the detection of alternative splicing. (DOCX 16.6 kb)
Additional file 6: Table S6. Transcriptome details for three transgenic lines with the most significantly reduced YPC and non-transformed controls. (DOCX 18 kb)
Additional file 7: Table S7. Details of the differentially expressed genes (DEGs) consistent in all three transgenic lines. (XLSX 82.6 kb)
Additional file 8: Table S8. Major metabolic pathways and candidate genes associated with *Psy1* down-regulation. (XLSX 10.1 kb)
Additional file 9: Table S9. Summary of silent mutations in *Psy1* identified by TILLING. (XLSX 11.7 kb)

## Abbreviations

DEGs: Differentially expressed genes
DPA: Days post anthesis
EMS: Ethyl methanesulfonate
GFP: Green fluorescent protein
GO: Gene ontology
NCBI: National Center for Biotechnology Information
PARSESNP: Project Aligned Related Sequences and Evaluate SNPs
*Psy1*: Phytoene synthase 1
qRT-PCR: Quantitative real-time PCR
RNAi: RNA interference
RNA-Seq: RNA sequencing
SIFT: Sorting Intolerant from Tolerant
TILLING: Targeting Induced Local Lesions IN Genomes
YPC: Yellow pigment content

## References

[1] Cuttriss AJ, Cazzonelli CI, Wurtzel ET, Pogson BJ, Rébeillé F, Douce R (2011). Carotenoids. Biosynthesis of Vitamins in Plants.

[2] Yeum KJ, Russell RM (2002). Carotenoid bioavailability and bioconversion. Annu Rev Nutr.

[3] Harjes CE, Rocheford TR, Bai L, Brutnell TP, Kandianis CB, Sowinski SG, Stapleton AE, Vallabhaneni R, Williams M, Wurtzel ET, Yan JB, Buckler ES (2008). Natural genetic variation in *lycopene epsilon cyclase* tapped for maize biofortification. Science.

[4] Fraser PD, Bramley PM (2004). The biosynthesis and nutritional uses of carotenoids. Prog Lipid Res.

[5] Baublis A, Decker EA, Clydesdale FM (2000). Antioxidant effect of aqueous extracts from wheat based ready-to-eat breakfast cereals. Food Chem.

[6] Zhang W, Dubcovsky J (2008). Association between allelic variation at the *phytoene synthase 1* gene and yellow pigment content in the wheat grain. Theor Appl Genet.

[7] Gallagher CE, Matthews PD, Li F, Wurtzel ET (2004). Gene duplication in the carotenoid biosynthetic pathway preceded evolution of the grasses. Plant Physiol.

[8] Li F, Vallabhaneni R, Wurtzel ET (2008). *PSY3*, a new member of the phytoene synthase gene family conserved in the Poaceae and regulator of abiotic stress-induced root carotenogenesis. Plant Physiol.

[9] He XY, Zhang YL, He ZH, Wu YP, Xiao YG, Ma CX, Xia XC (2008). Characterization of phytoene synthase 1 gene (*Psy1*) located on common wheat chromosome 7A and development of a functional marker. Theor Appl Genet.

[10] Flavell RB, Bennett MD, Smith JB, Smith DB (1974). Genome size and proportion of repeated nucleotide sequence DNA in plants. Biochem Genet.

[11] Lawrence RJ, Pikaard CS (2003). Transgene-induced RNA interference: a strategy for overcoming gene redundancy in polyploids to generate loss-of-function mutations. Plant J.

[12] Travella S, Klimm TE, Keller B (2006). RNA interference-based gene silencing as an efficient tool for functional genomics in hexaploid bread wheat. Plant Physiol.

[13] Sestili F, Janni M, Doherty A, Botticella E, D’Ovidio R, Masci S, Jones HD, Lafiandra D (2010). Increasing the amylose content of durum wheat through silencing of the *SBEIIa* genes. BMC Plant Biol.

[14] Becker D, Wieser H, Koehler P, Folck A, Mühling KH, Zörb C (2012). Protein composition and techno-functional properties of transgenic wheat with reduced α-gliadin content obtained by RNA interference. J Appl Bot Food Qual.

[15] Dong ZY, Feng B, Liang H, Rong CW, Zhang KP, Cao XM, Qin HJ, Liu X, Wang T, Wang DW (2015). Grain-specific reduction in lipoxygenase activity improves flour color quality and seed longevity in common wheat. Mol Breeding.

[16] Bleeker PM, Spyropoulou EA, Diergaarde PJ, Volpin H, De Both MTJ, Zerbe P, Bohlmann J, Falara V, Matsuba Y, Pichersky E, Haring MA, Schuurink RC (2011). RNA-seq discovery, functional characterization, and comparison of sesquiterpene synthases from *Solanum lycopersicum* and *Solanum habrochaites* trichomes. Plant Mol Biol.

[17] Slade AJ, Fuerstenberg SI, Loeffler D, Steine MN, Facciotti D (2005). A reverse genetic, nontransgenic approach to wheat crop improvement by TILLING. Nat Biotechnol.

[18] Stemple DL (2004). TILLING–a high-throughput harvest for functional genomics. Nat Rev Genet.

[19] Nadolska-Orczyk A, Przetakiewicz A, Kopera K, Binka A, Orczyk W (2005). Efficient method of *Agrobacterium*-mediated transformation for triticale (X. *Triticosecale* Wittmack). J. Plant Growth Regul.

[20] Livak KJ, Schmittgen TD (2001). Analysis of relative gene expression data using real-time quantitative PCR and the 2^−ΔΔCT^ Method. Methods.

[21] Zhai SN, He ZH, Wen WE, Jin H, Liu JD, Zhang Y, Liu ZY, Xia XC (2016). Genome-wide linkage mapping of flour color-related traits and polyphenol oxidase activity in common wheat. Theor Appl Genet.

[22] Zhou XH, Wang K, Lv DW, Wu CJ, Li JR, Zhao P, Lin ZS, Du LP, Yan YM, Ye XG (2013). Global analysis of differentially expressed genes and proteins in the wheat callus infected by *Agrobacterium tumefaciens*. PLoS ONE.

[23] Li H, Durbin R (2009). Fast and accurate short read alignment with Burrows-Wheeler transform. Bioinformatics.

[24] Langmead B, Trapnell C, Pop M, Salzber SL (2009). Ultrafast and memory-efficient alignment of short DNA sequences to the human genome. Genome Biol.

[25] Li B, Dewey CN (2011). RSEM: accurate transcript quantification from RNA-Seq data with or without a reference genome. BMC Bioinformatics.

[26] Ye J, Fang L, Zheng HK, Zhang Y, Chen J, Zhang ZJ, Wang J, Li ST, Li RQ, Bolund L, Wang J (2006). WEGO: a web tool for plotting GO annotations. Nucleic Acids Res.

[27] Zhang Y, Su J, Duan S, Ao Y, Dai JR, Liu J, Wang P, Li YG, Liu B, Feng DR, Wang JF, Wang HB (2011). A highly efficient rice green tissue protoplast system for transient gene expression and studying light/chloroplast-related processes. Plant Methods.

[28] Yoo SD, Cho YH, Sheen J (2007). *Arabidopsis* mesophyll protoplasts: a versatile cell system for transient gene expression analysis. Nat Protoc.

[29] Uauy C, Paraiso F, Colasuonno P, Tran RK, Tsai H, Berardi S, Comai L, Dubcovsky J (2009). A modified TILLING approach to detect induced mutations in tetraploid and hexaploid wheat. BMC Plant Biol.

[30] Ng PC, Henikoff S (2003). SIFT: predicting amino acid changes that affect protein function. Nucleic Acids Res.

[31] Taylor NE, Greene EA (2003). PARSESNP: a tool for the analysis of nucleotide polymorphisms. Nucleic Acids Res.

[32] Slade AJ, McGuire C, Loeffler D, Mullenberg J, Skinner W, Fazio G, Holm A, Brandt KM, Steine MN, Goodstal JF (2012). Development of high amylose wheat through TILLING. BMC Plant Biol.

[33] Wesley SV, Helliwell CA, Smith NA, Wang MB, Rouse DT, Liu Q, Gooding PS, Singh SP, Abbott D, Stoutjesdijk PA, Robinson SP, Gleave AP, Green AG (2001). Construct design for efficient, effective and high-throughput gene silencing in plants. Plant J.

[34] Holzberg S, Brosio P, Gross C, Pogue GP (2002). Barley stripe mosaic virus induced gene silencing in a monocot plant. Plant J.

[35] Miki D, Itoh R, Shimamoto K (2005). RNA silencing of single and multiple members in a gene family of rice. Plant Physiol.

[36] McGinnis K, Murphy N, Carlson AR, Akula A, Akula C, Basinger H, Carlson M, Hermanson P, Kovacevic N, McGill MA, Seshadri V, Yoyokie J, Cone K (2007). Assessing the efficiency of RNA interference for maize functional genomics. Plant Physiol.

[37] Dibari B, Murat F, Chosson A, Gautier V, Poncet C, Lecomte P, Mercier I, Bergès H, Pont C, Blanco A, Salse J (2012). Deciphering the genomic structure, function and evolution of carotenogenesis related phytoene synthases in grasses. BMC Genomics.

[38] Shewry PR, Underwood C, Wan YF, Lovegrove A, Bhandari D, Toole G, Mills ENC, Denyer K, Mitchell RAC (2009). Storage product synthesis and accumulation in developing grains of wheat. J Cereal Sci.

[39] Decourcelle M, Perez-Fons L, Baulande S, Steiger S, Couvelard L, Hem S, Zhu CF, Capell T, Christou P, Fraser P (2015). Combined transcript, proteome, and metabolite analysis of transgenic maize seeds engineered for enhanced carotenoid synthesis reveals pleotropic effects in core metabolism. J Exp Bot.

[40] Xu Q, Yu KQ, Zhu AD, Ye JL, Liu Q, Zhang JC, Deng XX (2009). Comparative transcripts profiling reveals new insight into molecular processes regulating lycopene accumulation in a sweet orange (*Citrus sinensis*) red-flesh mutant. BMC Genomics.

[41] Enfissi EMA, Barneche F, Ahmed I, Lichtlé C, Gerrish C, McQuinn RP, Giovannoni JJ, Lopez-Juez E, Bowler C, Bramley PM (2010). Integrative transcript and metabolite analysis of nutritionally enhanced *DE-ETIOLATED1* downregulated tomato fruit. Plant Cell.

[42] Pan ZY, Zeng YL, An JY, Ye JL, Xu Q, Deng XX (2012). An integrative analysis of transcriptome and proteome provides new insights into carotenoid biosynthesis and regulation in sweet orange fruits. J Proteomics.

[43] Baker KE, Parker R (2004). Nonsense-mediated mRNA decay: terminating erroneous gene expression. Curr Opin Cell Biol.

[44] Zhu Y, Li Y, Chen Y, Li H, Liang H, Yue S, Zhang A, Zhang X, Wang D, Jia X (2005). Generation and characterization of a high molecular weight glutenin 1Bx14-deficient mutant in common wheat. Plant Breed.

[45] Saito M, Nakamura T (2005). Two point mutations identified in emmer wheat generate null *Wx-A1* alleles. Theor Appl Genet.

[46] Sun YW, He ZH, Ma WJ, Xia XC (2011). Alternative splicing in the coding region of *Ppo-A1* directly influences the polyphenol oxidase activity in common wheat (*Triticum aestivum* L.). Funct. Integr. Genomic.

[47] Pandit J, Danley DE, Schulte GK, Mazzalupo S, Pauly TA, Hayward CM, Hamanaka ES, Thompson JF, Harwood HJ (2000). Crystal structure of human squalene synthase A key enzyme in cholesterol biosynthesis. J Biol Chem.

[48] Welsch R, Arango J, Bär C, Salazar B, Al-Babili S, Beltrán J, Chavarriaga P, Ceballos H, Tohme J, Beyer P (2010). Provitamin A accumulation in cassava (*Manihot esculenta*) roots driven by a single nucleotide polymorphism in a phytoene synthase gene. Plant Cell.

[49] Smith CJW, Chu TT, Nadal-Ginard B (1993). Scanning and competition between AGs are involved in 3′ splice site selection in mammalian introns. Mol Cell Biol.

[50] Howitt CA, Cavanagh CR, Bowerman AF, Cazzonelli C, Rampling L, Mimica JL, Pogson BJ (2009). Alternative splicing, activation of cryptic exons and amino acid substitutions in carotenoid biosynthetic genes are associated with lutein accumulation in wheat endosperm. Funct Integr Genomic.

[51] Rodríguez-Suárez C, Atienza SG, Pistón F (2011). Allelic variation, alternative splicing and expression analysis of *Psy1* gene in *Hordeum chilense* Roem. et Schult. PLoS ONE.

[52] Ahloowalia BS, Maluszynski M, Nichterlein K (2004). Global impact of mutation-derived varieties. Euphytica.

