# Supplementary material for: Genetic analysis of phytoene synthase 1 (Psy1) gene function and regulation in common wheat
Source: BMC Plant Biol. 2016 Oct 21;16:228. doi: 10.1186/s12870-016-0916-z (PMC5073469; doi:10.1186/s12870-016-0916-z)
Supplement: Additional file 1: Table S1. — Primers used for the RNAi vector construction and positive transgenic line detection. (DOCX 16.7 kb) [file 12870_2016_916_MOESM1_ESM.docx]

**Additional file 1: Table S1** Primers used for the RNAi vector construction and positive transgenic line detection.

| Primer name | Sequence (5’-3’) |
| --- | --- |
| PS-F | ATTAGGATCCATGGCCACCACCGTCACG |
| PS-R | GTGCTTCGAACGAGGTAGAAGGTCTTGGCGTA |
| PA-F | ATTAGGTACCATGGCCACCACCGTCACG |
| PA-R | GCGCGCTAGCCGAGGTAGAAGGTCTTGGCGTA |
| In-F | GCGAGGTACCGTAAGCCACTCACTCACTACCAATAC |
| In-R | GCGCGGATCCCTGGGAAATTATTCGAAACACC |
| FAD-F | ATTCTTATTTCTTTCCAGTAGC |
| FAD-R | AGAAGCGGCATAATGTGAGA |
