# Supplementary material for: Genetic analysis of phytoene synthase 1 (Psy1) gene function and regulation in common wheat
Source: BMC Plant Biol. 2016 Oct 21;16:228. doi: 10.1186/s12870-016-0916-z (PMC5073469; doi:10.1186/s12870-016-0916-z)
Supplement: Additional file 2: Table S2. — Culture media used in this study for callus induction and differentiation. (DOCX 16.7 kb) [file 12870_2016_916_MOESM2_ESM.docx]

**Additional file 2: Table S2** Culture media used in this study for callus induction and differentiation.

| Media | Compositions |
| --- | --- |
| Induction medium | MS mineral salt, 30 g L^-1^ sucrose, 1.0 mg L^-1^ thiamine HCl, 150 mg L^-1^ aspartic acid, 2.0 mg L^-1^ 2,4-dichlorophenoxyacetic acid (2,4-D), 2.4 g L^-1^ phytagel; pH 5.8 |
| Selection medium | Callus induction medium, MS vitamins, 10 mg L^-1^ geneticin (G418), 250 mg L^-1^ carbenicillin; pH 5.8 |
| Regeneration medium | MS basic medium, 30 mg L^-1^ sucrose, 0.2 mg L^-1^ 2,4-D, 0.1 g L^-1^ ascorbic acid, 25 mg L^-1^ G418, 250 mg L^-1^ carbenicillin; pH 5.8 |
