# Supplementary material for: Genetic analysis of phytoene synthase 1 (Psy1) gene function and regulation in common wheat
Source: BMC Plant Biol. 2016 Oct 21;16:228. doi: 10.1186/s12870-016-0916-z (PMC5073469; doi:10.1186/s12870-016-0916-z)
Supplement: Additional file 3: Table S3. — Primers developed for qRT-PCR analysis. (DOCX 17.5 kb) [file 12870_2016_916_MOESM3_ESM.docx]

**Additional file 3: Table S3** Primers developed for qRT-PCR analysis.

| Specificity | Name | Sequence (5’-3’) |
| --- | --- | --- |
| *Psy-all* | Psy1-all-F | GGGATGTCGGAGAAGATGCAAG |
|  | Psy1-all-R | AGAGAGGCCCAAACCGGCCACC |
| *Psy-A1* | Psy-A1-F | GGTGATGGGCATTGCGCCCGAC |
|  | Psy-A1-R | GATCTGCCTCTTCATGAATTTT |
| *Psy-B1* | Psy-B1-F | CATCGTCAAAGGAGTCGTCACT |
|  | Psy-B1-R | TAAGAGCAGCGATTTCCCGTAT |
| *Psy-D1* | Psy-D1-F | AAGAGGGAGGATATATTTGCCA |
|  | Psy-D1-R | ACAACAGAGAGGCCCAAAC |
| *β-actin* | Actin-F | CTGATCGCATGAGCAAAGAG |
|  | Actin-R | CCACCGATCCAGACACTGTA |
