# Supplementary material for: Genetic analysis of phytoene synthase 1 (Psy1) gene function and regulation in common wheat
Source: BMC Plant Biol. 2016 Oct 21;16:228. doi: 10.1186/s12870-016-0916-z (PMC5073469; doi:10.1186/s12870-016-0916-z)
Supplement: Additional file 4: Table S4. — Homoeolog-specific primers developed for mutation detection by TILLING. (DOCX 17.9 kb) [file 12870_2016_916_MOESM4_ESM.docx]

**Additional file 4: Table S4** Homoeolog-specific primers developed for mutation detection by TILLING.

| Homoeolog | Name | Sequence (5’-3’) | Length (bp) |
| --- | --- | --- | --- |
| *Psy-A1* | PA3 | F: CCGCCTGCTACCCAAGAAGAAA  R: GTCCAAACCAGGCATGGTGAAT | 570 |
| *Psy-A1* | PA4 | F: GCCCCTCGCAGCAACAGCAGCA  R: AATTATGCACCGGCTAAGCTGA | 1090 |
| *Psy-A1* | PA6 | F: TTTTGGTGTTTCGAATAATTTC  R: CTTCTCCCTTCCTTTGCCAGAA | 789 |
| *Psy-B1* | PB2 | F: GTCCCAACGCGTAGCACATC  R: CTGGTTCGCCAACCCGAGA | 964 |
| *Psy-D1* | PD6 | F: AAATTAGGCTTTTTGTTGGCTG  R: GGTTTATATGGGAAATTAGGCG | 853 |
| *Psy-D1* | PD8 | F: TGGAGAAGAGTAAGCAACTCAT  R: TATATACATCTTGTTCTGTCGT | 978 |
