# Supplementary material for: Genetic analysis of phytoene synthase 1 (Psy1) gene function and regulation in common wheat
Source: BMC Plant Biol. 2016 Oct 21;16:228. doi: 10.1186/s12870-016-0916-z (PMC5073469; doi:10.1186/s12870-016-0916-z)
Supplement: Additional file 5: Table S5. — Primers designed for the detection of alternative splicing. (DOCX 16.6 kb) [file 12870_2016_916_MOESM5_ESM.docx]

**Additional file 5: Table S5** Primers designed for the detection of alternative splicing.

| Mutant | Sequence (5’-3’) |
| --- | --- |
| M090122 | F:CCGCCTGCTACCCAAGAAGAAA |
|  | R:GCTCTCAGCTGTCGCCTTGGAG |
| M092201 | F:ATGGGCCATCTACGTGTGGTGT |
|  | R:GAGCTCAAAGGCAGATGTCGCG |
