# Supplementary material for: Genetic analysis of phytoene synthase 1 (Psy1) gene function and regulation in common wheat
Source: BMC Plant Biol. 2016 Oct 21;16:228. doi: 10.1186/s12870-016-0916-z (PMC5073469; doi:10.1186/s12870-016-0916-z)
Supplement: Additional file 6: Table S6. — Transcriptome details for three transgenic lines with the most significantly reduced YPC and non-transformed controls. (DOCX 18 kb) [file 12870_2016_916_MOESM6_ESM.docx]

**Additional file 6: Table S6** Transcriptome details for three transgenic lines with the most significantly reduced YPC and non-transformed controls.

| Sample | Clean reads | Unigenes | Differentially expressed genes (DEGs) | | |
| --- | --- | --- | --- | --- | --- |
|  |  |  | Total | Up-regulated | Down-regulated |
| 273-2A | 12,905,930 | 1,128,107 | 948 | 820 | 128 |
| 275-3A | 12,866,862 | 1,160,285 | 930 | 853 | 77 |
| 279-1A | 12,890,492 | 1,192,915 | 992 | 804 | 188 |
| CK | 12,885,928 | 1,228,996 |  |  |  |

CK, non-transformed controls
